# Scales and Historical Evolution: Methods to Reveal the Relationships between Ecosystem Service Bundles and Socio-Ecological Drivers—A Case Study of Dalian City, China

**DOI:** 10.3390/ijerph191811766

**Published:** 2022-09-18

**Authors:** Xiaolu Yan, Xinyuan Li, Chenghao Liu, Jiawei Li, Jingqiu Zhong

**Affiliations:** 1 Center of Marine Economy and Sustainable Development, Key Research Base of Humanities and Social Sciences of the Ministry Education, Dalian 116029, China; 2 University Collaborative Innovation Center of Marine Economy High-Quality Development of Liaoning Province, Dalian 116029, China; 3 Institute of Marine Sustainable Development, Liaoning Normal University, Dalian 116029, China; 4 Faculty of Electronic Information and Electrical Engineering, Dalian University of Technology, Dalian 116029, China; 5 State Key Laboratory of Resources and Environmental Information System, Institute of Geographic Sciences and Natural Resources Research, CAS, Beijing 100101, China

**Keywords:** ecosystem service bundles, multiple temporal–spatial scales, dynamic evolution, driving factors

## Abstract

Ecosystem service (ES) bundles can be defined as the temporal and spatial co-occurrence of ESs. ES bundles are jointly driven by socio-ecological factors and form at different scales. However, in recent research, a few studies have analyzed the dynamic evolution and driving mechanisms of ES bundles at different scales. Therefore, this study explored the spatial patterns of six ESs supplied in Dalian (China) from 2005 to 2015 at three spatial scales, determining the distribution and evolution patterns of ES bundles and their responses to socio-ecological driving factors. Our results are as follows: (1) We identified four ES bundles representing ecological conservation, water conservation, ecological depletion, and food supply. The developmental trajectory of each ES bundle could be attributed to the combined effects of environmental conditions and urban expansion. In particular, the water conservation bundle and food supply bundle were changed to the ecological depletion bundle. Given the ongoing urbanization, the conflict between ESs has intensified. (2) The impact of socio-ecological driving factors on ES bundles vary with scale. At three spatial scales, the digital elevation model (DEM) and normalized difference vegetation index (NDVI) had a great impact on ES bundles. Urbanization indicators also strongly explain the spatial distribution of ES bundles at the county and grid scales. The interaction factor detector shows that there is no combination of mutual weakening, indicating that the formation of ES bundles is driven by multiple factors in Dalian. Overall, this study used a more holistic approach to manage the ecosystem by studying the temporal-spatial dynamics of the multiple ESs.

## 1. Introduction

Since the 1940s, coastal areas have undergone rapid urbanization [1]. Although the rapid urbanization has greatly contributed to the economic development of coastal areas, in turn, it has negatively affected human well-being and the sustainable development of urban ecosystems [2]. A major challenge in ecosystem management is to cope with the conflicts between multiple ecosystem services (ESs) in complex socio-ecological systems [3,4,5]. This is because ESs are not independent and interact in complex ways [6,7,8,9]. ES bundles refer to “a collection of multiple ESs that co-occur in space and time” [3,10,11]. ES bundles emphasize the interdependence of ESs and provide a visual method for characterizing the complex nexus between multiple ESs [3,12]. Quantifying the relationships between multiple ESs is key to ensuring a sustainable supply of ESs and safeguarding human well-being.

The corpus of studies about ES bundles has rapidly evolved in the last decade, as one can visualize multiple ESs to provide intuitive information about environmental management [8,11,13,14,15]. The commonly used evaluation methods include principal component analysis (PCA) and cluster analysis (CA) [16,17,18]. With advancements in the understanding of ES bundles, scholars have introduced the self-organizing map (SOM) method to quantify the distribution of ES bundles [19,20,21]. SOM advantageously combines the capacities of dimensionality reduction with cluster-analysis capabilities and, due to this, is widely recognized in environmental science [22,23]. Currently, the application of ES bundles is mainly focused on ES trade-offs and synergies [24], regionally dominant ESs, ecological-function zoning [25], landscape planning and management [10], etc. Previous studies have promoted our understanding of ES bundles, but have not considered their dynamic evolution due to the significant temporal–spatial heterogeneity of ESs [9,26]. The identification and analysis of multiyear ES bundle evaluations can be used to reveal the ES spatial pattern more effectively [14,16]. To reduce the uncertainty, it is, therefore, necessary to explore the evolution trajectory of ES bundles. Changes in the trajectory of ES bundles can be addressed for more user-friendly identification of the evolutionary characteristics of urban ecosystems [9]. In turn, it will foster the prevention of further expansion of ecological-conflict areas.

The relationships among multiple ESs are often affected by socio-economic and environmental factors, such as climate [27], urbanization rate [28], and land-use change [7]. Some scholars have discussed the formation mechanism of the nexus among varied ESs from the perspective of driving factors. For instance, Lamarque, et al. (2014) [27] explored the impact of climate change on the interaction of ESs in the Alpine grassland. Furthermore, Peng, et al. (2020) [14] used the decoupling index to explore the impact of socio-economic factors on the interaction of urban ESs. In brief, most scholars have mainly addressed the influence of natural environmental factors or urbanization indicators on the interaction of multiple ESs, while the common driving effect of natural and socio-economic factors has remained understudied [29,30]. Potential conflicts among multiple ESs can be identified by analyzing the compound influence of socio-economic and natural factors on ES bundles [29].

Urban landscape is highly heterogeneous and dynamic; a variety of ESs are produced by various ecological processes and structures [8,9]. Each process is focused at different spatial scales [31]. As the scale changes—which is determined by the ecological processes of ES bundles—types and spatial distribution patterns may also change [32], and the final recommendations for land-use management may also differ [8], implying that research should be conducted across different spatial scales. Moreover, socio-economic factors show scale effects [33]; additionally, benefit distribution and ES management occur at specific scales [32,34,35]. Currently, studies on different ES bundles and their driving mechanisms are mainly analyzed at a single scale [8,9]. These studies mainly focus on specific scales such as watershed scale, administrative scale, and grid scale. However, regardless of the degree of scale dependence, the impact of socio-ecological driving factors on ESs is often inconsistent in different studies [31]. Research on the dynamic changes of multi-scale ES bundles and their driving mechanisms is still lacking, and providing effective information for decisionmakers of different units to formulate ecosystem management policies is challenging [34,36]. Thus, with an aim of providing clear and relevant information about ESs, the scale of different ES processes should consider research and assessment. To clarify the overall characteristics of the spatial distribution of various ESs in the region at a rough scale—and to identify the formation mechanism of ES bundles at a fine scale—can not only clarify the inherent laws of the formation of ESs, but also help managers at all levels to diagnose the ecosystem in a timely manner and optimize regulation measures to achieve sustainable ES management, which is of great significance to improve human well-being [35,37,38].

Dalian is the epitome of China ’s coastal-regional development model and one of the earliest coastal cities in the history of China [39]. However, industrial pollution, claiming land from the sea, and other human activities have triggered the continuous loss of ecological land, habitat loss, and the degradation of the ecosystem’s supply capacity [40]. All these factors have seriously undermined the resource base for sustainable development in Dalian. From this standpoint, studying the relationships between the spatial distribution of multiple ESs and socio-ecological driving factors will help implement effective ecological management in coastal areas in the context of rapid urbanization. To this end, we use the coastal city of Dalian in China as a case study. Specifically, the main objectives of this study were to: (1) explore the spatial distribution of ESs and ES bundles at three spatial scales over time, from 2005 to 2015; and (2) explore the impact of socio-ecological driving factors on ES bundles at different scales.

Dalian is the epitome of China ’s coastal-regional development model and one of the earliest coastal cities in the history of China [39]. However, industrial pollution, claiming land from the sea, and other human activities have triggered the continuous loss of ecological land, habitat loss, and the degradation of the ecosystem’s supply capacity [40]. All these factors have seriously undermined the resource base for sustainable development in Dalian. From this standpoint, studying the relationships between the spatial distribution of multiple ESs and socio-ecological driving factors will help implement effective ecological management in coastal areas in the context of rapid urbanization. To this end, we use the coastal city of Dalian in China as a case study. Specifically, the main objectives of this study were to: (1) explore the spatial distribution of ESs and ES bundles at three spatial scales over time, from 2005 to 2015; and (2) explore the impact of socio-ecological driving factors on ES bundles at different scales.

## 2. Materials and Methods

### 2.1. Study Area

**Figure 1:**
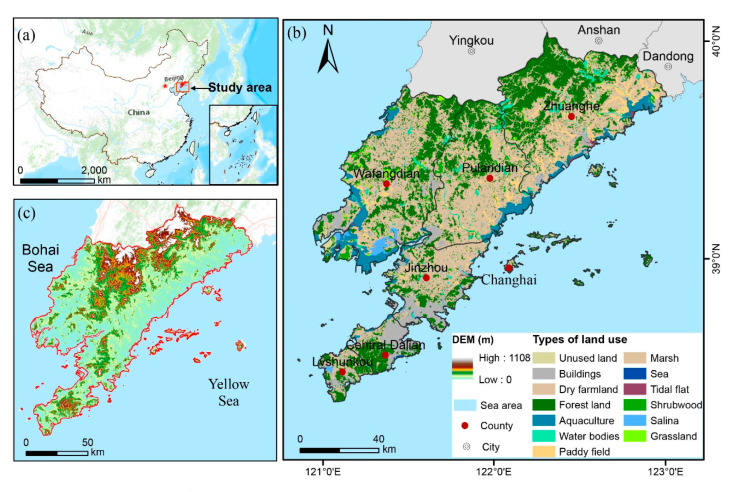
Study area in China (**a**), topography (**b**), and land-use classification in 2015 (**c**).

Dalian (120°98′–123°52′ E, 38°73′–40°22′ N) is located on the east coast of the Eurasian continent, facing North Korea, South Korea, and Japan across the sea (Figure 1a). Its administrative area is 12,573.85 km^2^, including four counties (Wafangdian, Pulandian, Zhuanghe, and Changhai) and three districts (Dalian Center, Jinzhou, and Lvshunkou) (Figure 1b). The area is dominated by a mountainous and hilly topography, with an altitude of up to 1108 m (Figure 1c) and a warm temperate continental monsoon-influenced climate with maritime characteristics. The average annual precipitation is 550–950 mm, which falls primarily in summer, and the average annual temperature is 10.5 °C. Over the past few decades, farmland and forestland have been the dominant land-use types in the study area (Figure 1b). However, due to the impact of high-intensity human activities, ecological land sharply decreased. It has subsequently caused increased fragmentation of habitats and increased soil erosion, posing a huge threat to the supply of ESs. Therefore, the restoration and protection of Dalian’s ecosystem is essential for the sustainable development of the regional ecological economy.

### 2.2. Conceptual Framework

**Figure 2:**
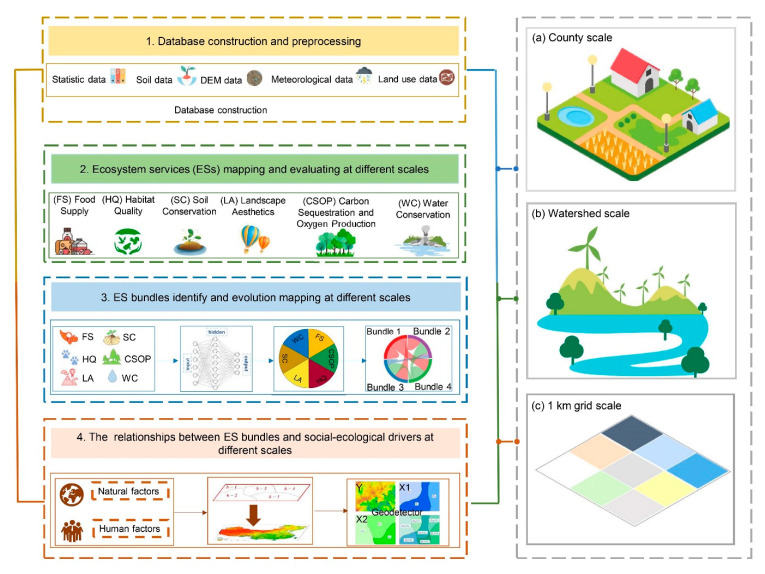
Conceptual research framework.

To explore the ES bundles and their connection with socio-ecological factors, we established a framework using the steps shown in (Figure 2). First, we built a database. Second, we mapped six ESs at different temporal–spatial scales, including food supply (FS), water conservation (WC), carbon sequestration and oxygen production (CSOP), soil conservation (SC), habitat quality (HQ), and landscape aesthetics (LA) at different temporal-spatial scales. Third, we identified the ES bundles, while also analyzing their dynamic trajectory changes at different temporal–spatial scales. Finally, we explored the linkages between socio-ecological driving factors and ES bundles at different scales.

### 2.3. Data Sources

We describe the datasets used in this study in Table 1. Note that all datasets were projected to the same coordinate system (WGS_1984_UTM_Zone_51N).

### 2.4. Mapping ESs at Multiple Spatial Scales

This study uses the following three criteria to determine the ESs indicators in Dalian: (1) First, ESs can reflect the current natural conditions in Dalian. Surrounded by sea on three sides and with numerous islands, Dalian is an important water-conservation/biological habitat. The diversity of the landscape also promotes the spread of aesthetics. (2) They are in accordance with the representations of different ecosystem service categories in the classification framework of the Millennium Ecosystem Assessment [8], including provisioning services, regulating services, supporting services, and cultural service. (3) ESs can express the current economic and food-security situations [41]. The planting and breeding industry is an industrial pillar of Dalian. With the advancement of urbanization, the population of Dalian is increasing, and the demand for food is also increasing. On this basis, six ecological service indicators were selected and evaluated, including: one provisioning service (FS), two regulating services (CSOP, WC), two supporting services (SC, HQ), and one cultural service (LA). We quantified ESs at the 1 km scale, and then calculated the average of ESs at the watershed and county scales. Table 2 provides the evaluation method for each ES in this study. Most explorations of ESs focus on specific scales, however, the provision and distribution of ESs may be different at different scales. Therefore, the three scales selected in this paper respectively represent the natural environment characteristics and the administrative management scale of Dalian. The 1 km scale is suitable for the precise management of ecosystems. As a natural geographical unit, the watershed scale can accurately reflect the biophysical characteristics of different ecosystem services, and has inherent advantages in solving the mismatch between ecological-process scale and human management. The county scale often represents the smallest scale of local landscape management, and is also the basic scale for landscape spatial planning and management decisions [8,9,31].

### 2.5. Identify ES Bundles at Multiple Spatial Scales

In this study, the self-organizing map (SOM) method, an unsupervised clustering algorithm with an artificial neural network, was applied to identify the ES bundles [17,49]. SOM algorithms reduce high-dimensional data by grouping observations based on their similarities while preserving the topological properties of the input data [19]. To identify and map the ES bundles, we initially performed Z-score standardization on six ESs to avoid excessive distance differences, driven by different units. Note that too many homogeneous regions would weaken the differences between different regions [50,51], while too few homogeneous regions would weaken the similarity within each homogeneous region. To identify the optimal cluster and to obtain more robust results, we determined the optimal number of clusters under three different spatial scales according to the ClusGap function in the R programming language. Finally, based on the selforgmap function of the Matlab R2019b platform, we experimented with different iteration times to provide the most suitable cluster-mapping results under different spatial scales.

### 2.6. Identification of Drivers of ES Bundles

The formation of and changes in ES bundles are closely related to environmental and socio-economic factors. Geodetector has advantages for studying heterogeneity of categorical variables and provides technical support for identifying the influencing factors of bundles. Due to this, it has been widely used in geography, ecology, and environmental studies [52,53]. The core idea of Geodetector is that if there is significant spatial consistency between the independent variable X and dependent variable Y, the association between them does exist. We divide these factors into two categories and briefly describe them in Table 3.

Geodetector can also be utilized for exploring the degree-of-interpretation of the dependent variable when two independent variable factors work together. The superposition of the two influencing factors may weaken or enhance the impact on the distribution of the ES bundles. The types of interactions are listed in Table 4.

## 3. Results

### 3.1. Spatial Distribution Characteristics of ESs at Different Spatial Scales

**Figure 3:**
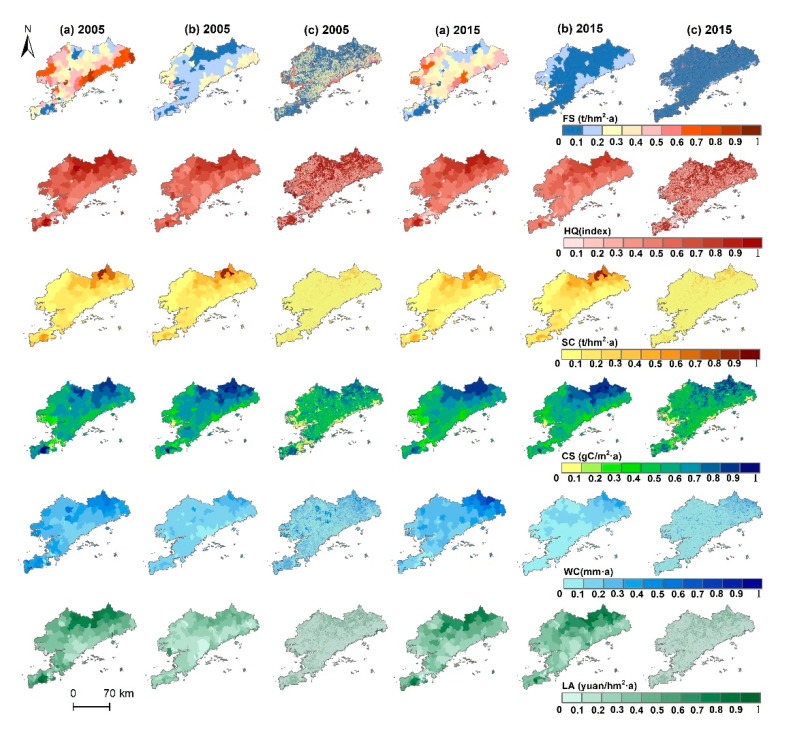
Spatial patterns of ESs in Dalian at three scales of analysis; (**a**) county scale, (**b**) watershed scale, (**c**) 1 km grid scale.

From 2005 to 2015, the spatial distribution of six ESs changed in different ways, and their spatial-pattern differences were shown at three scales (Figure 3). The provisioning service (FS) was mainly the harvest of grain and aquatic products in Dalian. At the grid scale, the high-value areas of food supply were distributed in the paddy fields and aquaculture areas along the eastern and western coastal margins, but at the watershed scale, the high-value areas were obviously smooth. The agglomeration of such high-value areas was more obvious at the county scale. The spatial distribution of CSOP showed similarities at the three analysis scales. Northeast and southwest of Dalian are high-altitude areas with high forest coverage. Therefore, the high-value areas related to CSOP and NDVI were mainly distributed in the northeast and southwest regions. The distribution patterns of SC, LA, HQ, and CSOP were similar, with high value areas concentrated in the mountainous regions along the northeast and southwest. The rougher the scale, the more evident the spatial clustering of high values in northeast and southwest Dalian is. In general, the dispersion of the spatial distribution of ESs increased with the refinement of the spatial scale. In terms of time, except for CSOP, the five ESs in the study area showed a significant decrease in 2015. Affected by reclamation, the area of non-ecological land continued to expand; the most significant degradation occurred in the east and west tidal flats and offshore.

### 3.2. Historical Patterns and Dynamics of ES Bundles at Different Spatial Scales

**Figure 4:**
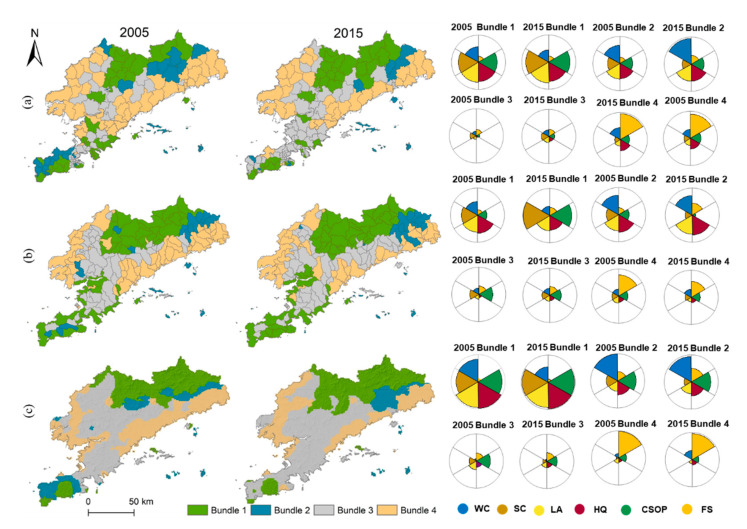
Spatial distribution of ES bundles in Dalian at the three scales of analysis from 2005 to 2015. Bundle 1–4 represents the bundles of ecological conservation, water conservation, ecological depletion, and food supply, respectively; (**a**) county scale, (**b**) watershed scale, (**c**) 1 km grid scale.

The SOM algorithm showed that the ES bundles from 2005 to 2015 were stabilized at three spatial scales (Figure 4), corresponding to the four ES bundles with similar social–ecological conditions and sets of ESs, namely: ecological conservation, water conservation, ecological depletion, and food supply. In this study, the spatial patterns of each ES bundle varied across different years. Specifically, ecological conservation bundle demonstrated high ecological benefits, characterized by a relatively uniform supply of CSOP, SC, WC, HQ, and LA, but featured lower FS. This finding can be related to the higher altitudes, which have less human interference and increased forest cover. It is concentrated in the forest areas of Zhuanghe and Pulandian in the north of the study area and in the Lvshunkou District in the south. At the county and watershed scales, spatial distribution of the ecological conservation bundle was different, but the dominance of forest-based services persisted; in the meantime, it has become more concentrated at the county and watershed scales. The water conservation bundle was concentrated in Zhuanghe in the east, Wafangdian in the west, and Lvshunkou District in the south, where the precipitation is relatively high. It is characterized by its ability to conserve water, while the supply of WC, HQ, and LA was relatively balanced at the watershed scale. The ecological depletion bundle exhibited the lowest ES ecological benefit; ES bundles of different scales were generally consistent, characterized by a low ability to provide all ESs. This is mainly because the ecological depletion bundle was concentrated in the central urban areas where districts and counties feature intense anthropogenic activities such as construction and land change. The food supply bundle was clustered in Zhuanghe in the east and Wafangdian in the west, characterized by its outstanding ability to provide FS, while the other five ESs were relatively weak, which was consistent with the grain supply bundle observed at different scales. This pattern could emerge due to the location of the food supply bundle as it was located at a low altitude with sufficient rainfall and superior irrigation conditions, which were more suitable for aquaculture and planting. In general, grid-scale analysis can provide a finer detail to ecosystem management information than county and watershed scales.

**Figure 5:**
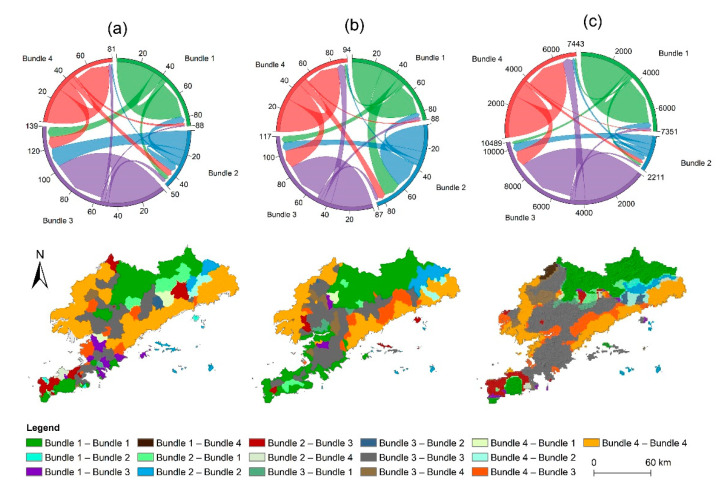
Dynamic changes of ES bundles in Dalian from 2005 to 2015; (**a**) county scale, (**b**) watershed scale, (**c**) 1 km grid scale.

At different scales, the spatial distribution of ES bundles changed obviously over time (Figure 5). The changes of ES bundles mainly followed two different trajectories, which were related to the natural environment and the process of urbanization. In general, the spatial distribution of the ecological conservation bundle was relatively invariable, being determined by the special terrain and, less-so, by the relatively low human interference and/or favorable forest coverage. The number of water conservation bundles has been greatly reduced at all three scales, and most of them are transformed into the ecological exhaustion bundle. At the county and grid scales, this change mainly occurs in the Lvshun New City in the southwest and the Huayuankou Economic Zone in Zhuanghe City in the east. At the watershed scale, it was mainly concentrated in Wafangdian in the west. Simultaneously, rapid urbanization has caused vegetation degradation. This phenomenon has greatly aggravated ecological damage and forced the ecological depletion bundle to become the dominant bundle in the region, which was consistent between different scales. The food supply bundle has also been significantly reduced at different scales. Some farmland areas were exposed to construction activities, and the food supply bundle has been transformed into the ecological exhaustion bundle. As an intermediate conclusion, it can be stated that promoting ecological restoration and moderating human activities can ensure the supply of several ESs.

### 3.3. Determining Socio-Ecological Drivers for ES Bundles at Different Spatial Scales

**Figure 6:**
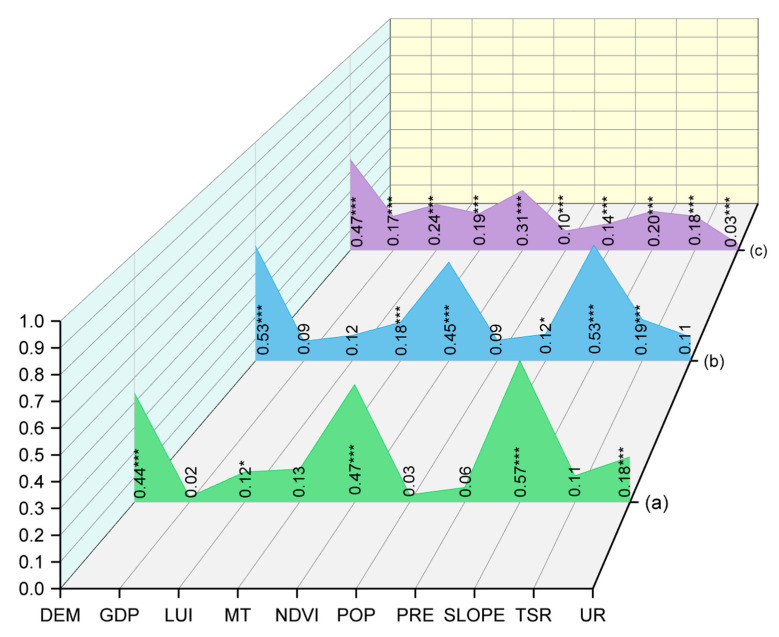
Factor detector results; *** means that q value is significant at the 0.001 level; * means that q value is significant at the 0.05 level; (**a**) county scale, (**b**) watershed scale, (**c**) 1 km grid scale.

The dynamic changes of ES bundles were determined by the socio-economic and environmental characteristics of the study area. The relative importance of each independent factor to the spatial distribution of ES bundles was determined by calculating the q value (Figure 6). Moreover, the explanatory power of ES bundles may vary at different scales. We used 2015 as a temporal scale; at the county scale, SLOPE, NDVI, DEM, and UR were the dominant socio-ecological driving factors, explaining 57%, 47%, 44%, and 18% of the distribution of ES bundles, respectively. At the watershed scale, SLOPE, DEM, NDVI, and TSR were the dominant social and ecological driving factors, which explained 53%, 53%, 45%, and 19% of the distribution of ES bundles, respectively. At the grid scale, DEM, NDVI, LUI, and MT were the dominant social and ecological driving factors, which explained 47%, 31%, 24%, and 19% of the distribution of ES bundles, respectively. In a word, DEM and NDVI have always had a strong influence on the spatial pattern of ES bundles as the scale changed. As a central city along the coast of China, Dalian is greatly affected by anthropogenic activities. From this perspective, UR was a direct manifestation of the local social and economic development. When human demand is in conflict with an ecosystem (such as through urban expansion and sea reclamation), short-term irreversible impacts on the ecosystem emerge. Meanwhile, DEM and NDVI are predictors of vegetation growth. Therefore, researching and formulating ecological and/or environmental protection-planning schemes for different ES bundles can more effectively maintain good ecological benefits.

**Figure 7:**
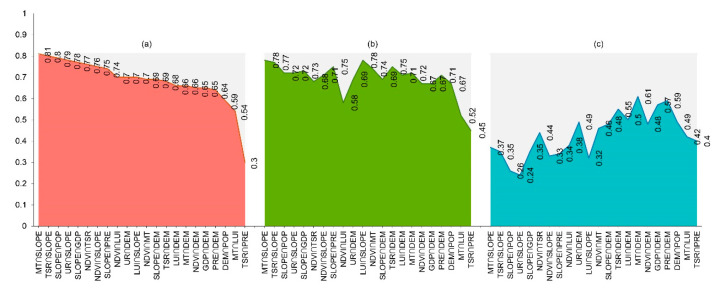
Results of factor interaction in Dalian from 2005 to 2015; (**a**) county scale, (**b**) watershed scale, (**c**) 1 km grid scale.

The impact of different combinations of historical driving factors on ES bundles should be considered, as well. We selected the 10 pairs of combinations that have the largest impact on the common driver of ES bundles at different scales (Figure 7). These combinations were dominated by double-factor enhancement, which indicated that the spatial differentiation of ES bundles was driven by multiple factors in Dalian. At the county scale, (MT∩SLOPE) had the largest explanatory power (q = 0.81), followed by (TSR∩SLOPE) (q = 0.80) and (SLOPE∩POP) (q = 0.79). At the watershed scale, (MT∩SLOPE) had the largest explanatory power (q = 0.78), followed by (LUI∩SLOPE) (q = 0.78) and (TSR∩SLOPE) (q = 0.77). At the grid scale, (MT∩DEM) had the largest explanatory power (q = 0.61), followed by (PRE∩DEM) (q = 0.59) and (GDP∩DEM) (q = 0.57). The relationships between different driving-factor combinations and ES bundles vary with scale. The strongest combination of driving factors at different scales was representative of the connection between different natural factors, suggesting that the natural factors were critical for the formation of ES bundles. Furthermore, at the county and grid scales, the interaction between socio-economic factors and natural factors also showed strong explanatory power.

## 4. Discussion

### 4.1. Model Validation and Uncertainty Analysis

As the model simulations are sensitive to changes in spatial scale and input data, model simulation results need to be validated [54]. It is not negligible that the robustness of ES evaluation directly affects subsequent interactions between different ESs. Model simulation results can be verified in two ways. One is to identify the relationship between observed and predicted values, and the other is to compare results with previous similar studies [55,56].

**Figure 8:**
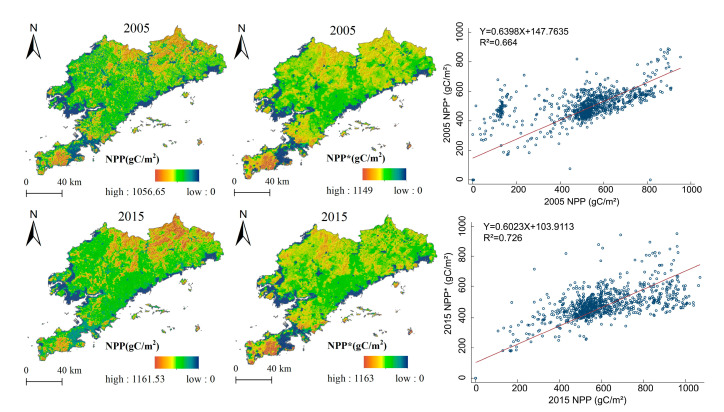
Comparison between NPP and NPP*; X: independent variable NPP; Y: dependent variable NPP*; R^2^: coefficient of determination. R^2^ between 0–1. the closer to 1, the better the regression fitting effect.

This study validates the results of four ESs simulated by the model, including water conservation, carbon sequestration and oxygen production, soil conservation, and habitat quality. First, the average water production in the period was retrieved by using the InVEST model for 2005–2015. The simulation revealed the average water production (3.79 km^3^/yr), which slightly exceeded the actual water volume (3.45 km^3^/yr) in the area as announced by the Liaoning Water Resources Department (http://slt.ln.gov.cn/, accessed on 25 March 2021). It might be due to an error of the spatial interpolation method, but the overall error is small. We established 1,000 random points in the ArcGIS model and extracted the NPP simulated by the CASA model (NPPCASA) and the MODIS17A3 data (NPPMODIS) published on the USGS official website in the same year (http://lpdaac.usgs.gov/products/mod17a3hv006/, accessed on 26 March 2020). We performed a linear regression analysis of the NPP that extracted the same points. As shown (Figure 8), there was a significant correlation between NPPCASA and NPPMODIS. The coefficients of determination (R^2^) were 0.664 and 0.726 in 2005 and 2015, respectively. This finding indicates that the CASA model has a higher simulation accuracy in the study area. We found that the average soil erosion modulus released by the Dalian Water Affairs Bureau (https://swj.dl.gov.cn/, accessed on 25 March 2021) was 23.60 t/ha in 2015, which was not significantly different from the actual soil erosion amount of 26.49 t/ha as calculated based on the RUSLE model. The habitat-quality model yielded results within the same range of values as those from northeast China. Namely, it revealed a habitat-quality value of 0.8–1.0 in the north and south of Dalian, and 0.0–0.2 in Central Dalian [40]. As seen prior, the simulation results are close to the empirical estimates, suggesting that the results are credible.

Although the evaluation model can reflect the supply capacity of ESs in Dalian to a large extent, there are still uncertainties in the ES evaluation of this study. For example, in some cases, the water-yield module overestimates surface runoff and ignores intra-annual variability in groundwater flow. Additionally, complex underlying geographies may have intricate water-balance processes [57], but the InVEST model cannot simulate the required parameters in complex terrain. In terms of habitat quality, this study failed to obtain appropriate measurements or statistics to validate the results of the InVEST model. In the future, more-representative indicators should be further explored and verified. While the InVEST model provides good estimates of regional water production, soil conservation, carbon sequestration and oxygen production, and habitat quality, the simplicity of the model and the lack of extensive observational data [54] add to the uncertainty of the study results [58]. Therefore, future research should strengthen the observation and verification of field test data, and use the measured experimental data to calibrate the model parameters to obtain greater confidence in the quantitative output [59], and further verify the model output.

### 4.2. Influencing Mechanism of ES Bundles

**Figure 9:**
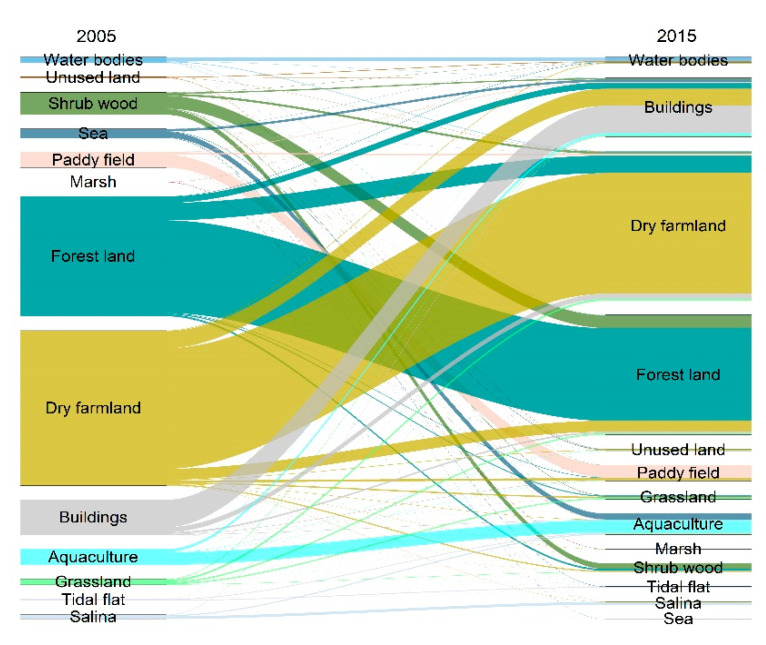
Land-use changes in Dalian from 2005 to 2015.

Comprehensive and in-depth assessment of how social–ecological driving factors impact the spatial distribution and cross-scale relationships of ES bundles were essential for the sustainable provision of ESs effectively [38,60,61]. Our results indicated that socio-ecological factors jointly drive the spatial distribution and evolution of ES bundles at three scales. DEM and NDVI were the most influential driving factors, which could explain the spatial distribution of ES bundles at three spatial scales in Dalian. Previous studies have found that areas with high altitude and vegetation coverage offered high supplies of supporting, regulating, and cultural services [30,42]. Our study confirmed these findings and further demonstrated the persistence and durability of the cross-scale impacts of DEM and NDVI on ES bundles. However, the socio-ecological factors might differ from the impacts of only the social–ecological factors on ES bundles at different scales. At the county and grid scales, the impact of UR and LUI on ES bundles could not be ignored either. LUI and UR are the key pressures of the urban social ecosystem; land-use change will have an important impact on the evolution of ES bundles. Understanding how land-use changes affect the relationships between ESs is a prerequisite condition for future coastal area planning and land-use management [7,62,63]. From 2005 to 2015, rapid urbanization has strongly affected the land coverage in the study area. Most impacts emerged in forested and offshore areas, which play an important role in providing biological protection and landscape aesthetics. Marine environments are ideal for tourism and recreational activities. In recent years, land reclamation in coastal areas has caused the destruction of coastal natural terrain, which has transformed many natural landscapes into human landscapes, thereby often reducing the aesthetic value of the landscape and damaging biodiversity. This explains why regulating, supporting, and cultural services are greatly affected by the process of urban expansion. Accordingly, we constructed a land-use transfer map (Figure 9), which can more-intuitively reflect the transfer direction and evolution process among land-use types. These results showed that over the past 10 years, the main changes in land-use types in Dalian have been the mutual conversion of forest land and cultivated land; the alteration of forest land and cultivated land to built-up land; and the change of sea to aquaculture land and built-up land. Notably, the areas of forest land, farmland, and sea decreased by 3.6%, 1.5%, and 2.4%, respectively, in 2005–2015. Meanwhile, the built-up and aquaculture land increased by 6.1% and 1.5%, respectively. Notably, built-up-area expansion is an important driver of the decrease in forest and cultivated land. Moreover, 56.21% of the built-up area came from cultivated land and 23.9% stemmed from forest land, indicating that the expansion of built-up land mainly came from the occupation of cultivated land and forest. The latter areas are concentrated in Lvshunkou and Jinzhou in the south. It should be emphasized that 66.55% of the transferred aquaculture land originated from the sea. Furthermore, poorly implemented reclamation projects may damage disaster prevention and mitigation facilities in coastal areas, ultimately affecting social stability and the sustainability of development. In short, policymakers and managers should recognize the relationships between different ecological communities and the ESs that they provide, while facilitating economic development. This consideration is a single measure on a long way towards the onset of sustainable coastal ecosystems and the mitigation of related damages in coastal areas.

### 4.3. Guidelines for Landscape Planning and Management

Multi-scale understanding of the relationships between ES bundles and social–ecological drivers is required to make decisions effectively in the regional governance of ESs [31,33,34,64,65]. As mentioned, Dalian is the epitome of China’s coastal regions. With the rapid economic development of Dalian, population density in the study area has also rapidly increased. Urban expansion causes a decrease in the fragmentation and natural ecological area of the ecosystem; this damages the services, while also harming people’s survival, security, social relationships, and health [66]. For example, urban infrastructure replaces landscape communities in natural areas. The spatial pattern characteristics of ES bundles persist in a multi-scale range, which is beneficial for ensuring the formulation of governance actions at different scales and reducing the unpredictability that might indirectly affect ESs [31]. Therefore, we concluded that the implementation of management interventions for specific scales might have a more significant effect on the provision of ESs.

Here we discuss the implications for landscape planning and management in Dalian and in similar coastal areas by using the results of this study. It is worth noting that ES bundle 1 focused on regulating and supporting cultural services, and the area was classified as an ecological protection area. Considering that the formation of ES bundle 1 is closely related to natural driving factors such as DEM and NDVI, we recommend that protection work should be carried out at the grid scale. In the future, all construction activities should be permanently prohibited to maintain the original functions of the land and prevent the expansion of mountainous cities [67]. Simultaneously, rational use of organic food processing should be developed using natural mountain resources within the ecological conservation bundle to reduce conflicts with regulating, supporting, and cultural services. ES bundle 2 specialized in water conservation; it has been clearly reduced over the past ten years, mainly due to the change in precipitation. We recommend that protection and restoration work be carried out at the watershed scale, strengthening the protection of the water-source areas of the Zhuang River, Biliu River, and Yingna River in the region [68]. Further restoration measures have to be taken in the damaged areas. Planting forests to protect WC from declining and nature-based solutions (such as sponge cities) can be incorporated into urban planning to improve regional water-conservation capabilities [9]. ES bundle 3 exhibited the state of exhaustion at different scales and continued to expand. The ecological exhaustion bundle was mainly located in the urban construction area, which should be restored and compensated in the future. The construction of the Dalian Changxing Island Lingang Industrial Zone, Lvshunkou Western Lingang New City, and Huayuankou Economic Zone from 2005 to 2015 has significantly changed the balance of aquatic and terrestrial ecological communities in this area. We suggest that governance should be conducted at the county level, so as to facilitate the formulation of corresponding policies by regional managers. Urban green belts should be considered in the future to expand the proportion of green space, thus protecting the area from the loss of urban ESs and providing considerable ecological benefits [6,69]. Simultaneously, it should be underlined that the reasonable development of offshore and tidal flat wetlands can form a balanced natural-coast, breeding-coast, and life-coast utilization pattern. It is also desirable to further promote carbon reduction and to offset actions within and outside urban boundaries to achieve carbon neutrality. In addition, ES bundle 4 is dedicated to food-supply services, which have also decreased over the past 10 years, mainly due to the construction and occupation of cultivated land. Protecting agricultural areas is essential for urban food security; thus, governmental actions should be undertaken at three spatial scales [5,70]. In the future, intensive agricultural production should be encouraged to reduce the number of abandoned farmlands and to develop the lower–middle farmland [9]. In addition, coastal and natural wetlands gradually evolve into artificial wetlands, such as aquaculture, which may cause further damage to the ecosystem [71]. In the future, the technology level of aquaculture should be improved, a transition from extensive production modes to energy-saving production modes made, coastal ecological corridors established, and landscape types enriched. In general, the emergence of potential conflicts between various ESs in Dalian critically depends on the specific management tools aimed at the landscape [10]. China nowadays is actively promoting the protection of coastal areas [28], and strives to realize the vision of harmonious coexistence between humans and nature. Therefore, we appeal to decisionmakers to incorporate the spatial distribution and historical dynamics of ES bundles into urban planning more broadly. This will deepen understanding of the versatility of ecosystems in environmental management and prevent conflict between ESs and beneficiaries.

### 4.4. Limitations and Future Directions

Our study offered a multi-scale and comprehensive approach to untangle the relationships among ES bundles and socio-ecological drivers at different spatial scales. We provided a deeper understanding about which areas have to be focused on conservation, transformation, and restoration, and provided insights into landscape planning and management. Our findings contribute to enriching the current knowledge on the scale-dependent changes of ecosystem management, and provide insights on integrating the scale-dependency of ESs into the governance of ESs to promote sustainability. The conclusion provides a user-friendly guideline for ecological management decision-making in Dalian and similar coastal cities. However, some potential limitations have to be acknowledged in this study. We only considered the bundling of the ES supply and have not considered the temporal and spatial aggregation of ES demand; however, ESs are closely related to human well-being. Identifying ES bundles by combining the supply and demand of ESs can better reflect the relationships between human development and the potential of ecosystems, especially in urban areas [5,10,14]. In addition, the high-resolution grid unit is conducive to the fine-grained management of decisionmakers according to local conditions [56]. However, undetected uncertainties could emerge due to spatial resampling of the used data, and this grid-scale-related error propagation was not taken into account [19]. Therefore, the formation mechanism of ES supply and demand bundles should be further explored in the future, and more-practically linked to the government decision-making process, so as to better guide ecosystem management measures and achieve the goal of harmonious human development.

## 5. Conclusions

In order to have in-depth understanding of the scale-dependence between ES bundles and socio-ecological driving factors, this study explored the spatial distribution, bundles, and driving factors of ESs at different temporal and spatial scales in Dalian. It is of great significance to promote the supply of diverse ecosystems and regional sustainable development. We found that the landscape pattern changed significantly in the context of rapid urbanization. In particular, WC, FS, HQ, SC, and LA exhibited a downward trend in 2015. To elucidate the ecological conflicts, we also examined the spatial distribution of ES bundles. This investigation found that the ES bundles’ patterns at different spatial scales were consistent. By integrating the identified major influencing factors of ES bundles at different spatial scales, we can propose feasible zoning and design options to facilitate sustainable ecosystem planning, bridging the gaps in previous theoretical and practical approaches to planning. According to the results of the ecological function bundles, Dalian can be divided into four landscape-function areas: an ecological protection area, water conservation area, ecological depletion area, and food supply area. The evolutionary trajectory of ES bundles is driven by social and ecological factors. DEM and NDVI can explain most of the variation in ES bundles at different scales, but UR and LUI can cause large variation in the spatial distribution of ES bundles at county and grid scales. Despite these challenges, natural and socioeconomic factors should be properly accounted for. We have shown that the historical dynamics of ES bundles deserve more attention. Integrating ecological and social drivers in ecosystem assessment can provide reasonable spatial information for the formulation of ecological management objectives in different regions. According to the evolution of ES bundles and the factors which influence it, the corresponding land-use planning is proposed to protect the sustainable development of Dalian. The results show that the ecological protection scheme of ES bundle 1, the water-source protection scheme of ES bundle 2, the ecological management scheme of ES bundle 3, and the agricultural and fishery optimization scheme of ES bundle 4 have good effects on improving the ecological environment. Therefore, the landscape-function zoning in this study can provide a spatial reference for Dalian’s ecosystem protection and urban planning.

## Figures and Tables

**Table 1:** Data sources for ES evaluation.

Data Category	ES Types						Data Source	Usage Details and Resolution
	FS	HQ	SC	LA	CSOP	WC		
Land-use data	√	√	√	√	√	√	Land-use data was obtained by interpreting Landsat TM/ET/OLI data from the USGS website (accessed on 3 November 2020) (https://usgs.gov/landsat, accessed on 5 December 2019)	Interpreted and obtained thirteen types of land-use in Dalian from 2005 to 2015 (30 m × 30 m)
MODIS data					√		Normalized difference vegetation index (NDVI) (http://lpdaac.usgs.gov/products/mod13q1v006/, accessed on 15 March 2020) Net primary production (NPP) (http://lpdaac.usgs.gov/products/mod17a3hv006/, accessed on 26 March 2020)	NDVI (MODIS13Q1 dataset) (250 m × 250 m)
								NPP (MODIS17A3 dataset) (500 m × 500 m)
Digital Elevation Model (DEM) data			√			√	Geospatial data cloud site (http://www.gscloud.cn/, accessed on 14 December 2019)	Based on the digital elevation model (DEM) data, extracted the slope and slope length by hydrology modeling (30 m × 30 m)
Soil data			√			√	China soil map based on harmonized world soil database (HWSD) (v1.1) (http://www.ncdc.ac.cn/portal/, accessed on 25 January 2020)	Including current data on silt, clay, sand, and organic carbon (1 km × 1 km)
Meteorological data			√		√	√	China meteorological data network (http://data.cma.cn/, accessed on 15 January 2020)	Includes meteorological data such as precipitation, evaporation, average temperature, wind speed, and solar radiation from thirteen weather stations in and around Dalian (Text data–daily and monthly)
Socio economic data	√			√			Statistical yearbook of Dalian (http://stats.dl.gov.cn/, accessed on 20 November 2020)	Including annual food production, tourism income in the study area (Text data–yearly)

**Table 2:** Indicators and methods used to measure each ES.

ESs	Description	Unit	Evaluation Methods and Key References
FS (Provisioning services)	Crops (cereals, fruits, vegetables), livestock products (meat, eggs, milk), aquatic products (shrimp, crab, fish)	(t/hm^2^·a)	Food yield per unit area is assigned to the corresponding land-use grid [42,43].
HQ (Supporting services)	Distribution of habitat quality was quantified by combining the sensitivity of the landscape type and the intensity of external threats	Index (0–1)	based on the habitat quality module in the integrated valuation of ecosystem services and trade-offs (InVEST) model [44].
SC (Supporting services)	Quantification of the supply of soil conservation caused by vegetation through the effect of vegetation on reducing soil loss and sediment accumulation	(t/hm^2^·a)	Use of the revised universal soil loss equation (RUSLE) model to estimate potential soil erosion and actual soil erosion [28].
CSOP (Regulating services)	Use of NPP data based on the principle of photosynthesis, in which 1 unit of organic matter can fix 1.63 units of carbon dioxide and production 1.2 units of oxygen	(g C/m^2^·a)	Estimation of NPP based on Carnegie–Ames–Stanford Approach (CASA) model [45,46].
WC (Regulating services)	Adoption of the principle of water balance, and calculate the flow rate coefficient, soil permeability, soil conservation, and hydraulic conductivity of Dalian to obtain water conservation	(mm·a)	Use of the InVEST model to quantify water yield [47,48].
LA (Cultural services)	Considering that the tourism industry can indirectly reflect landscape aesthetics, tourism income is used to characterize the service value of landscape aesthetics	(yuan/hm^2^·a)	The equivalent value per unit area method was used to assign the revised tourism revenue per unit area to the landscape category.

**Table 3:** Driving factors selected for this study.

Category	Driving Factors	Spatial Resolution	Source
Natural factors	PRE—annual average precipitation	1 km × 1 km	http://data.cma.cn/, accessed on 15 January 2020
	MT—mean temperature	1 km × 1 km	http://data.cma.cn/, accessed on 15 January 2020
	TSR—total solar radiation	1 km × 1 km	http://data.cma.cn/, accessed on 15 January 2020
	NDVI—normalized difference vegetation index	1 km × 1 km	https://www.nasa.gov/, accessed on 15 March 2020
	SLOPE—terrain slope	1 km × 1 km	http://www.gscloud.cn/, accessed on 16 December 2019
	DEM—digital elevation model	1 km × 1 km	http://www.gscloud.cn/, accessed on 14 December 2019
	CLAY—percentage of clay in soil	1 km × 1 km	http://westdc.westgis.ac.cn, accessed on 25 January 2020
	OM—percentage of organic matter in soil	1 km × 1 km	http://westdc.westgis.ac.cn, accessed on 25 January 2020
	SAND—percentage of sand in soil	1 km × 1 km	http://westdc.westgis.ac.cn, accessed on 25 January 2020
	SILT—percentage of silt in soil	1 km × 1 km	http://westdc.westgis.ac.cn, accessed on 25 January 2020
Human factors	POP—population density	1 km × 1 km	http://www.resdc.cn/, accessed on 20 November 2020
	UR—urbanization rate	1 km × 1 km	http://www.resdc.cn/, accessed on 20 November 2020
	GDP—GDP per unit area	1 km × 1 km	http://www.resdc.cn/, accessed on 20 November 2020
	LUI—land-use intensity	1 km × 1 km	https://usgs.gov/landsat, accessed on 20 November 2020

**Table 4:** Type of interaction.

Judgment Criteria	Type of Interaction
q(X1∩X2) < Min(q(X1), q(X2))	Non-linear reduction
Min(q(X1), q(X2)) < q(X1∩X2) < Max(q(X1), q(X2))	Single-factor nonlinearity reduction
q(X1∩X2) > Max(q(X1), q(X2))	Double factor enhancement
q(X1∩X2) > q(X1) + q(X2)	Non-linear enhancement
q(X1∩X2) = q(X1) + q(X2)	Independent

## Data Availability

All data and codes are available via the corresponding author.
